# Bioimpedance Phase Angle as a Prognostic Tool in Late-Onset Pompe Disease: A Single-Centre Prospective Study With a 15-year Follow-Up

**DOI:** 10.3389/fcell.2022.793566

**Published:** 2022-02-18

**Authors:** Sabrina Ravaglia, Rachele de Giuseppe, Annalisa Carlucci, Susan Jehne, Grazia Crescimanno, Lara Ahmad, Matteo Paoletti, Gabriele Clemente, Anna Pichiecchio, Rosella Bazzano, Serena Cirio, Enza Maria Valente, Cesare Danesino, Paola De Filippi, Alice Tartara, Hellas Cena

**Affiliations:** ^1^ IRCCS Mondino Foundation, Pavia, Italy; ^2^ Laboratory of Dietetics and Clinical Nutrition, Department of Public Health, Experimental and Forensic Medicine, University of Pavia, Pavia, Italy; ^3^ Pneumologia Riabilitativa, IRCCS Istituti Clinici Scientifici Maugeri, Pavia, Italy; ^4^ Dipartimento di Medicina e Chirurgia, Università dell’Insubria, Varese, Italy; ^5^ Oberhavel Kliniken—Marwitzer Hennigsdorf, Hennigsdorf, Germany; ^6^ Italian National Research Council, Institute for Biomedical Research and Innovation, Palermo, Italy Regional Center for Prevention and Treatment of Respiratory Complications of Rare Genetic Neuromuscular Diseases, Villa Sofia-Cervello Hospital, Palermo, Italy; ^7^ Department of Molecular Medicine, IRCCS Policlinico San Matteo, University of Pavia, Pavia, Italy; ^8^ Clinical Nutrition and Dietetics Service, Unit of Internal Medicine and Endocrinology, ICS Maugeri IRCCS, Pavia, Italy

**Keywords:** late-onset pompe disease, enzyme replacement therapy, nutritional assessment, long-term effectiveness, outcome

## Abstract

**Background:** Late-onset Pompe disease (LOPD) is an autosomal-recessive metabolic myopathy caused by deficiency of the lysosomal enzyme Acid Alpha—Glucosidase (GAA), leading to glycogen accumulation in proximal and axial muscles, and in the diaphragm. Enzyme Replacement Therapy (ERT) with recombinant GAA became available in 2006. Since then, several outcome measures have been investigated for the adequate follow-up of disease progression and treatment response, usually focusing on respiratory and motor function. Prognostic factors predicting outcome have not been identified till now.

**Methods:** In this single Centre, prospective study, we evaluate the response to enzyme replacement therapy in 15 patients (7 males) with LOPD in different stages of disease, aged 49.4 ± 16.1, followed-up for 15 years. Treatment response was measured by the 6-min walking test, vital capacity in supine and upright position, respiratory muscle strength, muscle MRI, manual muscle testing. We investigated the usefulness of Body Impedance Vectorial Analysis for serial body composition assessment.

**Results:** Although most patients with LOPD benefit from long-term treatment, some secondary decline may occur after the first 3–5 years. Some nutritional (lower body mass index, higher fat free mass, higher phase angle) and disease parameters (higher creatinine and shorter disease duration at the beginning of treatment) seem to predict a better motor outcome. Lower Phase Angle, possibly reflecting loss of integrity of skeletal muscle membranes and thus treatment mis-targeting, seems to correlate with worse treatment response on long-term follow-up.

**Conclusion:** Body Impedance Vectorial Analysis is a fast, easily performed and cheap tool that may be able to predict long-term treatment response in patients with LOPD. Low Phase angle may serve as a marker of muscle quality and may be used to predict the response to a muscle-targeted intervention such as ERT, thus improving the identification of patients needing a closer follow-up due to higher fragility and risk of deterioration.

## Introduction

Late-Onset Pompe disease (LOPD) (OMIM #232300) is an autosomal-recessive lysosomal disease caused by deficiency of acid alpha-glucosidase (GAA). Although glycogen accumulation occurs in several tissues, the phenotype is a progressive myopathy, mainly involving girdles, axial muscles, and the diaphragm (Chan et al., 2017).

Since 2006, with the availability of Enzyme Replacement Therapy (ERT), several reports have addressed the identification of the proper measure to assess disease progression and treatment response. The ideal measure should be sensitive to little changes and independent of subjective collaboration. The long-term effectiveness of ERT is another unsolved issue: longitudinal studies agree on a positive response in most patients in the first 1–3 years of treatment (Schoser et al., 2017), but only few data on long-term effectiveness are available.

Disease management usually includes nutritional assessment, in view of the potentially detrimental effects of both overweight (increasing respiratory/skeletal muscle work) and undernutrition (reflecting worsening of pulmonary function and predisposing to respiratory infections). Body Impedance Vectorial Analysis (BIVA) is a tool for non-invasive, indirect assessment of fat-free-mass (FFM), fat mass (FM), body cell mass (BCM), and Phase Angle (PhA) (Calcaterra et al., 2019). PhA is an index of nutritional status; it is used as a prognostic index of survival in several diseases (Calcaterra et al., 2019; Garlini et al., 2019).

In this prospective, single-centre study, we evaluated the long-term effectiveness of ERT in a population of LOPD in different stages of disease. The assessment included serial examination of respiratory function, muscle strength, walking abilities, muscle MRI, and nutritional parameters.

## Methods

Diagnosis of Pompe disease relied on low GAA levels and was confirmed by genetic analysis. All genetic analyses were conducted in a single center (Authors: CD, PdF); DNA was extracted by routine methods using the “GENE ELUTE” kit by Sigma. Amplification of all GAA exons and their flanking region was performed; sequence reactions were performed with the ABI PRISM Big Dye Terminator Cycle Sequencing Kit (Applied Biosystems Washington, UK). Mutations were confirmed by sequencing independent PCR products. We graded *GAA* mutations according to their functional consequences on GAA activity (http://www.pompevariantdatabase.nl) (Supplementary Table S1).

We assessed prospectively*,* since the beginning of ERT (T0), at least yearly:

- blood chemistry*:* creatinine, creatine kinase (CK), albumin, lipid profile
- Anti-rhGAA IgG: at T0, then every year during the first 5 years of ERT, then each time a clinical variation or an adverse reaction occurred. Antibody search was performed according to standard procedures (ELISA) and confirmed by a radio-immunoprecipitation (RIP) assay, by Genzyme Clinical Specialty Lab, Corp. (Framingham, MA 01560-1227)
- muscle strength: by quantitative handheld dynamometry (CITEC, The Netherlands) (Spink et al., 2010) of bilateral thigh adductors, knee flexors, knee extensors; all tests were made by the same neurologist (Author SR).
- muscle endurance: by the 6-min walk test (6MWT). Absolute values were adopted for within-subjects comparisons through different time-points; the percentage of the expected value according to sex, age, height, weight, was also calculated (Troosters et al., 2002)
- respiratory function, by: a) Forced vital capacity (FVC) and FVC% in sitting/supine position; b) ΔVC (“postural drop”), calculated as (FVC upright-FVC supine)/FVC upright: ≥20% was considered as an index of diaphragmatic weakness; these examinations were performed between 8:30 and 11.00 a.m. in triplicate, with patients wearing a nose clip and breathing through a flange-type mouthpiece. For each parameter and each position, the highest value of three technically satisfactory measurements was used. c)Maximal Inspiratory (MIP) and maximal expiratory (MEP) mouth pressures, measured in upright seated and in supine position; patients were strongly advised to produce maximum inspiratory (Mueller maneuver) at near residual volume for MIP, and expiratory (Valsalva maneuver) effort at total lung capacity for MEP. All tests were repeated at least three times and the best value was recorded. All pulmonary function measurements were performed according to the standards of ATS/ERS (American Thoracic Society, 2002).
- disease stage, by Walton score (Ravaglia et al., 2010).

We assessed, every 18–28 months**:**

- muscle MRI of thigh muscle, with calculation of the sum Mercuri score (Carlier et al., 2011) of anterior, posterior, medial thigh (the same muscles assessed by dynamometry); all measures were performed in blind, by two radiologists (Authors GC, MP) and discussed with a third experienced radiologist (AP) in case of discordant findings.
- Body composition, assessed by Body Impedance Vectorial Analyisis (BIOSMART, Eupraxia srl, Italy), by applying an alternating electric current at 800 microA and fixed 50-Hz rate frequency; measurements were performed as described (Calcaterra et al., 2019). BIVA measures resistance and reactance; after entering age, sex, height, weight, predictive equations estimate FM (indicating water-free body component), FFM (representing the remaining body components: skeletal muscle tissue, internal organs, interstitial fat), BCM (reflecting metabolically active tissue and intracellular water), PhA (proportional to the ratio of reactance, related to FFM, and resistance, depending on BCM). PhA normal value for adults is expressed in degrees; normal value is 6–8° (range 3–15°); since values vary with sex and age, we calculated Z-scores: (patient value–mean value of controls matched for age and sex)/standard deviation. FM, FFM, and BCM were expressed as percentage of bodyweight and according to age and sex (https://biodyncorp.com/pdf/quick_start_guide_310.pdf).
- Exclusion criteria were: a) contraindications to BIVA: implanted pacemaker, pregnancy, breast-feeding; b) “severe” disease, conditioning the reliability of the outcomes, defined as: FVC <30%, invasive ventilation, inability to walk at least 60 m on the 6MWT. Details on patients with severe disease (*n* = 6), who were excluded from the analysis of outcome measures, are in Supplementary Table S2. The clinical status of the first Italian adult receiving ERT through Expanded Access Programs in 2005 (patient 19 in Supplementary Table S2 and in Supplementary Figure S1) has been described in a previous report with a 3-year follow-up (Ravaglia et al., 2008).

### Statistical Analysis

Results are expressed as median/interquartile range, or mean/standard deviation (SD), depending on the distribution of the variable. Longitudinal analyses of outcomes utilized generalized linear models. For the analysis of prognostic predictors, continuous variables were dichotomized, using the median as cut–off. We used Spearman/Pearson tests for linear correlations, and Wilcoxon test for dependent variables, Kruskal-Wallis, or ANOVA, depending on the distribution of the variable, for between-groups comparisons. Paired-sample tests were used to evaluate the outcomes longitudinally. We annotated any major event occurring during follow-up (need for walking aids, ventilator support, death) and its timing (year after ERT start) and performed survival analysis by entering any presumed prognostic variable as cited above; continuous variables, again, were transformed into categorical by dividing them and utilizing the median as the cut-off. Significance was considered for *p* ≤ 0.05. For statistical analysis, we used SPSS version 25.

## Results

### Baseline Features

Demographic, clinical, genetic features of the patients, their disease course and main disease–related events are in Supplementary Figure S1 and in Table 1. The functional severity of the *GAA* mutation did not influence age at onset (*p* = 0.418). Baseline worse 6MWT% was associated with longer disease duration (rho = −0.7, *p* = 0.015). Supplementary Table S3 shows baseline 6MWT and FVC of each patient, the year when ERT was started, the occurrence and timing of main respiratory/motor events during ERT. Supplementary Tables S4, S5 show 6MWT and FVC over time. All patients had been compliant to ERT during the follow-up, and did not require any temporal discontinuation due to concomitant diseases or other complications. Some delay in ERT infusion (even 30 days) had occurred, in coincidence with supplying difficulties, during the first 6 months of treatment only, and only for patients not coming from the Lumbardy region. After that period, patients always had regular infusion except for occasional delays in holidays times, not exceeding 6–8 days.

**TABLE 1:** Main demographic and disease features of 18 patients on ERT.

	Patients (*n* = 18)
ERT follow-up duration, median (IQR, range) (y)	12 (8–14; 2–15)
Age at symptom onset, median (IQR, range) (y)	36 (26–48; 7–68)
Disease duration from symptom onset to start of ERT, median (IQR, Range) (y)	13 (10–21; 4–28)
Age at ERT start (IQR, range) (y)	53 (39.7–60.2; 28–82)
Sex M:F	7:11
6MWT% (12 patients, median, IQR, range)	67 ± 23 (IQR 52.75–89.5)
FVC% (12 patients, mean, DS, range)	73.8 ± 21.3 (IQR 61.75–90.25)
Genotype	
c.-32-13T>G	100% (18/18)
Second mutation
c.525delT	7/18
c.784G>A	2/18
c.2237G>A	2/18
others	7/18
Wheelchair/ventilation at the start of ERT (n)	
wheelchair + mechanical ventilation (invasive)	2 (1)**
wheelchair only	1*
mechanical ventilation only (invasive)	6 (1*)**
no wheelchair and no mechanical ventilation	9

*Patients with “very severe” disease belong to these groups (total * = 6), *y* = years, *n* = number of patients.

### Disease Course of Patients Treated With ERT

Eighteen patients began ERT between 2005 and 2012 and were followed-up for a median of 12 years. After excluding six patients with severe disease (separately described in Supplementary Table S2), we evaluated the outcomes of 12 patients with mean FVC 73.8 ± 21.3% and mean 6MWT 67 ± 23% at T0. Baseline 6MWT correlated with FVC (rho = 0.84, *p* = 0.001). Mean onset age was 37.3 ± 16.3 years (range 7–65). ERT was started at a mean age of 45.6 ± 11.7 years (28–60), thus with a mean latency of 12.9 ± 6.3 years (range 4–23) from symptoms onset.

**FIGURE 1:**
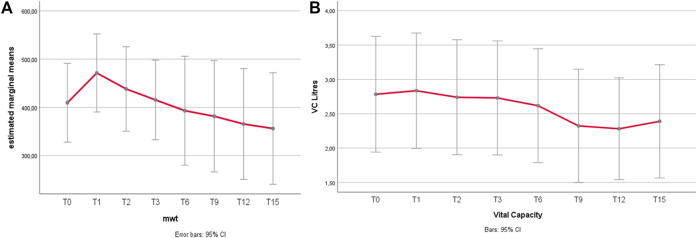
**(A,B)** 6MWT and FVC variations over time. **(A)** 6MWT after an improvement during the first year of treatment, the distance walked at the 6MWT declined gradually. Relative to the baseline, the mean distance walked increased from 367 to 424 m at 1 year (*p* = 0.010), with a return to baseline (mean 377 m) at 3 years, and a mild decline after 9 years (350 mt at T6 vs. 330 mt at T9, *p* = 0.282, n.s.; 350 mt at T9 vs. 314 m at T12, *p* = 0.007). Grey bars represent 95% confidence interval (CI). **(B)** FVC Unlike 6MWT, the FVC (forced vital capacity) does not show significant changes under treatment for most part of the follow-up duration. The only significant difference is a mild worsening trend of FVC between T6 and T9, being the only significant change (*p* = 0.011), with no significant improvement within the firsts 6 years, including the 1st year of treatment.

The main outcomes over time are in Figure 1 and details on individual FVC/6MWT variations over time are in Supplementary Tables S4, S5: a positive response at 1 year (T1) was not invariably associated to a better long-term outcome. A better outcome on the 6MWT was associated to higher creatinine (rho 0, 51, *p* = 0.048), higher CK (rho = 0.5, *p* = 0.046) and lower BMI (rho = −0.53, *p* = 0.040), while longer disease duration had a mild, non-significant negative impact (*p* = 0.065). There were no predictors of FVC outcome, and no correlation between respiratory outcome and motor outcome (ΔFVC and Δ6MWT: r = 0.454, *p* = 0.088), indicating possible inhomogeneous responses for motor and respiratory function.

Results of secondary outcomes are detailed in Supplementary Figures S2A–E. A postural drop > 20% was observed in 40% of patients at T0, and even doubled (80%) at the last follow-up. Muscle strength improved in the anterior thigh (knee extensors) only, and correlated with better baseline 6MWT (*p* = 0.033) and better final 6MWT (*p* = 0.008). Muscle MRI showed a progression of fatty degeneration, more evident at the posterior thigh. Anterior thigh lower MRI score correlated with better 6MWT, reflecting better walking ability.

All patients developed low titers of anti-rhGAA (1:100 to 1:1,600) antibodies, which did not seem to influence treatment response.

### Definition of ERT Responder vs. Non-Responder and Overall Outcome

The disease course shows high inter-individual variability, the response to treatment can change over time, and may be different with regard to different (respiratory vs. motor) outcomes (Supplementary Figure S3). Supplementary Table S6 compares three different scoring methods for outcome: a) the score proposed by Harlaar et al. (2019), also analyzing a population with a prolonged follow-up; b) the sum % changes in FVC and 6MWT; c) the occurrence of “major events” defined as a newly occurring change in motor or respiratory function, for instance, the need for walking or ventilatory support during follow-up.

We decided to adopt the occurrence of “major events,” and thus identified 5/12 patients (1, 5, 6, 9, 10) as “non-responders,” for the reasons detailed in Supplementary Table S6 legend, also fitting our clinical impression.

Survival analysis shows a positive effect of better initial 6MWT% (*p* = 0.033, with 6MWT dichotomized based on the median value of 53%) on the overall outcome, which is easily explained by considering that the ability to walk defines the final outcome with regard to “major events”. There was no significant effect of sex (*p* = 0.624), mutation severity (*p* = 0.796), disease duration before starting ERT (*p* = 0.771), initial FVC% (*p* = 0.495) or Walton score at T0 (*p* = 0.319) on the overall outcome (Table 2).

**TABLE 2:** Differences in basal nutritional parameters between responders and non-responders^a^.

	Responders (*n* = 7) Mean (SD)	Non-responders (*n* = 5) Mean (SD)	*p* value
Age	57.3 (15)	58.6 (16)	0.815
Disease duration	12.2 (4.9)	13 (6.5)	0.80000
Age at symptom onset	40.1 (16.4)	34.8 (19)	0.59000
Age at ERT start	48.3 (9)	47 (17.8)	0.87000
6MWT T0%	87.3 (21)	58.17 (24.1)	0.028
BMI T0	21.6 (4.3)	26.9 (7.4)	0.15400
FM% T0	21.4 (12.9)	31.6 (10.3)	0.1320
TBW/weight T0	2.9 (3.5)	−2.3 (6.5)	0.076
ECW/TBW T0	2.5 (3.9)	7.2 (4.7)	0.064
FFM% T0	80.8 (10)	68.3 (10.3)	0.0350
BCM%/FFM T0	52.1 (4.6)	46.3 (5.8)	0.053
Phase Angle (PhA), T0	5.5 (0.9)	4.7 (0.9)	0.076
Phase Angle Z-score, T0	−0.6 (0.9)	−1.3 (0.8)	0.04700
Delta PhA	−0.3 (0.6)	−0.5 (0.3)	0.607
FVC upright %, T0	79.3 (26.6)	73.3 (9.7)	0.669

ERT, enzyme replacement therapy; 6MWT, 6-min-walking test; BMI, body mass index; TBW, total body weight; ECW, extracellular water; FFM, fat free mass; BCM, body cell mass; PhA, phase Angle; FVC, forced Vital capacity; Delta PhA, difference between Phase angle at T0 and at the last follow-up.

a Definition of non-responder: occurrence of “major events” defined as the newly occurring need for walking or ventilator support during follow-up, after the beginning of ERT.

### Nutritional Assessment

Among blood chemistry tests, higher creatinine levels (rho 0.526, *p* = 0.044) correlated with FFM% (*p* = 0.009); serum triglycerides inversely correlated with FFM% (*p* = 0.036). Mean BMI was 23.7 ± 6.1 kg/m^2^ (range 14.2–41 kg/m^2^): according to the WHO classification^1^, one patient was severely obese (patient 6, BMI = 41 kg/m^2^), one was overweight (patient 4, BMI = 29.41), and one underweight (patient 12, BMI = 14.2). The underweight patient had normal FFM, thus not suggesting conditions such as cachexia, sarcopenia or protein malnutrition. BMI correlated inversely with FFM% (*p* = 0.000) and directly with FM% (*p* = 0.000). As expected, females had higher FM% (16.07 ± 7.3% males, 30.48 ± 12.5% females, *p* = 0.019), lower FFM% (84.7% males, 69.1% females, *p* = 0.011) and lower PhA (5.9 ± 0.83 males, 4.58 ± 0.48 females, *p* = 0.002). FFM% was below the reference range in patients 6, 10, 11; one of them, patient 6, showed co-existence of high BMI and low FFM, a condition labelled as “sarcopenic obesity” and regarded as an unfavorable prognostic sign.

FFM% correlated with 6MWT (*p* = 0.010), Δ6MWT (difference between 6MWT-T0 and final 6MWT, *p* = 0.033), and muscle strength (*p* = 0.037), but not with respiratory parameters (FVC, FVC%, and ΔVC). Higher baseline albumin correlated with better Δ 6MWT (*p* = 0.033).

PhA Z-scores at T0 ranged from −0.3 to −2.20 and did not correlate with baseline motor/respiratory function (Supplementary Tables S7, S8).

Higher PhA Z-score, FFM, BCM, all correlated with better outcome (Table 2). Supplementary Figure S4 shows PhA, FFM of each patient and their initial and final thigh MRI.

**FIGURE 2:**
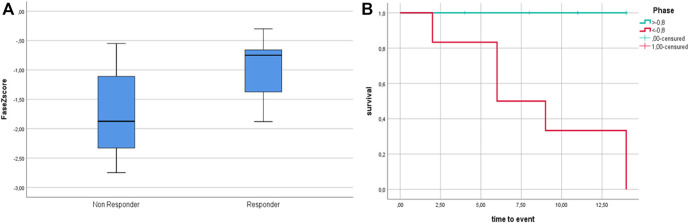
**(A)** PhA Z-score at T0 among non-responders and responders. **(B)**: Survival analysis according to dichotomized Phase Angle Z-score. Phase Angle is significantly lower in non—responders (*p* = 0.047); survival analysis according to dichotomized Z-scores (based on median Z-score cut-off = −0.8) show better outcome for patients with Z score > −0.8 (*p* = 0.016).

Follow-up PhA was assessed after 72 ± 34 months and showed a small but significant decrease over time (−0.1 ± 0.21°, *p* = 0.007). PhA Z-scores at T0 correlated with the final outcome (*p* = 0.045). Figure 2 shows PhA-Z scores in responders/non-responders, and survival analysis.

## Discussion

Although marketing authorization of Myozyme dates back to 16 years ago now, there are still few studies on its long-term effectiveness. We found that variations in individual response to ERT may occur over time; to furtherly complicate any judgement on treatment effectiveness, patients may behave differently as regards different outcomes (i.e., respiratory vs. motor).

### Main Motor and Respiratory Outcomes: 6MWT and FVC

The 6MWT improved during the first year of treatment in 58% of patients, then stabilized for 3–6 years, with a slow decline thereafter in 50% of patients (Supplementary Figure S3), especially in those with longer disease duration. This improvement in motor response peaking 1–3 years after the start of ERT, followed by stabilization, is common to most studies (Anderson et al., 2014; Kuperus et al., 2017; Schoser et al., 2017), and confirmed by recent reviews (Dornelles et al., 2021; Sarah et al., 2021). A recent review (Berli et al., 2021) failed to detect significant changes in FVC, that remained stable during a follow-up ranging 6 months to 7 years in different studies. However, reviews are generally biased by un-homogeneous follow-up durations.

The late decline in 6MWT we observed is in line with two others prolonged follow-up studies, meaning studies with at least a 5-to-10-years follow-up such as Harlaar et al. (2019), and Papadimas et al. (2021), this latter also monocentric, although retrospective, and including patients followed up for up to 11 years. These studies also showed that deterioration of motor over baseline may occur after the 6-years mark.

Upright FVC showed a prolonged stabilization, with a mild decline occurring only after the first 6 years of treatment in 5/12 (42%) patients. We did not collect pre-treatment FVC, but other studies have shown a steady, linear, 1.6–4.6% decline per year in untreated patients (Wokke et al., 2008; Van der Beek et al., 2009; van der Ploeg, 2010), so that detection of stabilization during such a prolonged follow-up can be considered as clinically meaningful, especially given that respiratory insufficiency is the major cause of death in LOPD.

By contrast, supine FVC continued to decline in most patients, with two of them even beginning non-invasive ventilation after the start of ERT. Similar observations are in line with other long-term follow-up studies (Kuperus et al., 2017; Harlaar et al., 2019), reporting upright FVC as stable during the first 5 years, then declining towards lower than baseline values at 10 years, and supine FVC declining despite ERT. It is possible that, since diaphragm weakness occurs earlier in the course of disease compared to the weakness of other thoracic respiratory muscles, it is more difficult to reverse its dysfunction. Another possible explanation may be that glycogen accumulation in spinal cervical motor neurons contributes to diaphragm weakness, and this may be unresponsive to ERT, since ERT does not pass the blood brain barrier (Turner et al., 2016).

### Predictors

In line with previous studies, we also failed to find robust prognostic factors for treatment response. However, higher creatinine values at the beginning of ERT, possibly reflecting preserved muscle mass (as also suggested by its correlation with FFM), and lower BMI, possibly associated with improved targeting of muscle tissue by the therapeutic enzyme in reduced FM (or simply with absence of the detrimental effects of overweight in a myopathy), seem to predict a better outcome in endurance measures such as 6MWT. On the contrary, respiratory outcome as assessed by upright FVC was not influenced by genetic, demographic, or disease parameters.

### Variations in Response Over Time

Concerning the temporal pattern of ERT response, with a better response during the first year of treatment, it is likely that, during the first year, there is improved clearance of glycogen from muscles, while, over time, these positive effects are outweighed by the negative effects of continued autophagic build-up, compromising the trafficking and processing of the therapeutic enzyme (Lim et al., 2018). Beyond autophagy, we also have to consider, in our series, as well as in other series with long follow-up, that physiological muscle aging (age-related decline in muscle mass and quality) likely occurs in patients older than 65. Indeed, as many as 50% of our patients are, actually, >65 at the final follow-up. In such a relatively old population, muscle satellite-cells activation is poor and muscle regeneration likely inadequate (Fry et al., 2015): indeed, with the exception of patient 6 (aged 41), all patients <65 showed a stable motor function, while secondary decline was more evident in patients older than 65 and with more prolonged disease duration.

### Definition of Responders vs. Non-Responders

When evaluating the effectiveness of a treatment, especially with continuous (non-ordinal) variables, the ideal outcome measure is the one that best captures a clinically meaningful change: the concept is distinct from “statistical significance.” The minimal clinically important difference (MCID) for changes in 6MWT varies from 14 to 30 m in different studies on pulmonary diseases and coronary artery diseases, while there are no analogue data on neuromuscular disorders (Bohannon and Crouch, 2017). In cardiorespiratory diseases, the mean baseline 6MWT varies from about 300 to 550 m, and thus the 14–30 m MCID variation translates in a percentage change from 3 to 10% compared to baseline. But cardiorespiratory diseases are not primary motor disorders, and thus the distance walked is expected to be more responsive to treatment compared to a myopathy, in view of the poor regeneration potential of a damaged muscle. Since our patients have a myopathy, baseline 6MWT is expected to be lower (and indeed mean baseline 6MWT in our series was 367 ± 148 m, range 60–580 m, 67 ± 23% of expected normal), and so we decided to consider a 30 mt or 10% variation from baseline as clinically meaningful for 6MWT.

For FVC, we choose a 200 ml or 10% variation to define a clinically meaningful change.

#### Longitudinal Variations in Response and Definition of Responder

At the individual level, 58.3% of patients had a significant initial improvement in either motor or respiratory outcome, or both, within the first year of ERT (Supplementary Figure S3). If we consider not only improvement, but also stabilization in one or both of the outcome measures as a positive effect, 100% of patients had at least some partial initial benefit from ERT during the first 3 years. Depending on the outcome measured, 50% (for 6MWT) to 58.3% (for FVC) of patients experienced a secondary decline. Still, at the final follow-up (median 12 years), 66.6% of patients had equal or better 6MWT and/or upright FVC compared to baseline (Supplementary Figure S3).

Thus, individual response to ERT may vary with time. This observation was also made by Harlaar et al. (2019), who also followed-up patients for a prolonged time and tried to measure ERT response by taking into account the individual disease course in a sort of ordinal grading for each patient.

A further problem in evaluating treatment response is that one patient may have a good respiratory outcome and a bad motor outcome and vice versa. Again, this could be solved by summing motor and respirator outcomes and taking as good responders the patients with better values. However, when we applied this method to our patients, we noticed that the final score failed to identify true bad vs. good responders. For instance, in Supplementary Table S6, patient 6 scores 7 due to preserved respiratory function, despite severe motor worsening (her status changed from able to walk to wheelchair bound); her response to treatment is thus clearly different, and clearly worse, compared to patient 11, who also scores badly due to initial worsening of both motor and respiratory function, but then she is relatively stable over time, and there are no substantial changes in her functional performances.

Therefore, after considering the limits of different scoring methods, we decided to consider as good responders those patients who, independent of the magnitude and persistence of the initial response, had no significant “major changes” in their motor (need for walking aids/wheelchair) or respiratory status (need for ventilator support, change in support type and duration of ventilation) over time. This method may underestimate small variations in patients in milder disease stages and in short follow-up studies, but we believe that it may be reliable through a prolonged follow-up, such as in our series, and may better reflect the patient’s functional status. Indeed, although the variations of 6MWT/FVC are informative, there are notable differences in terms of the ability to walk and the quality of breathing. We thus considered as “poor outcome” or non-responders those patients who changed their motor status (need for walking aids or wheelchair) or respiratory status (need for ventilator support or change in support type and duration) during the follow-up, and annotated the time to these changes.

One limitation of our study was lack of analysis of quality of life measures. At the time when the study was started (2005–2006), there were no specific quality of life measures for LOPD, and the most commonly adopted measure was SF-36. We early had the impression it was not appropriate for these patients, we realized that the patients completed the scores reluctantly and uncaring. Indeed, lack of quality of like measures is common to most reviewed studies (Schoser, Berli, Dornelles). In a very recent review (Dornelles et al., 2021), only 6 studies reported QL measures, and in all cases it was SF36; the changes observed were only in the physical domain, with substantial heterogeneity and very low certainty of evidence, suggesting that this scoring system may not be the best suitable measure for LOPD. Further outcomes are thus desirable, including scores more specific for LOPD or, at least, more specific to neuromuscular diseases, although evaluating a subjective measure in an open trial will remain, in my opinion, challenging: we see that, even when asked about subjective improvement, some patients (especially older patients) tend to report they are well, since they are aware of the costs of treatment and always fear the interruption of treatment if they admit a clinical worsening.

### Nutritional Assessment

#### Body Composition in Other Myopathies

BMI alone is not likely to capture the true nutritional status in LOPD, since it is not a good indicator of body composition, nor it is an indicator of the proportion of FM and FFM. Saure et al. (2018), by analyzing body composition in a population of Duchenne muscle dystrophy, observed that BMI may be normal despite an increase in FM%, and that this latter correlates with disease severity. The evaluation of skinfold thickness fails to detect this increase in FM, since subcutaneous adipose tissue is normal, and the FM increase is related to substitution of degenerating FFM by FM at the muscle level (Mok et al., 2010), and can be detected by BIA.

#### FFM and FM in Our Population of LOPD

LOPD is a metabolic disease affecting several tissues, but the phenotype is essentially that of a myopathy, thus—like Duchenne—is expected to subvert muscle structure, as confirmed by MRI studies (Ravaglia et al., 2010). However, variation in muscle structure in LOPD is not generalized, at least in mild to moderate disease stages, but rather confined to certain muscle groups: thus, it is not known whether muscle degeneration in LOPD can cause detectable variations in body composition, and whether these variations correlate with motor function (allowing them to be used for disease staging and, if sensitive enough to little changes, for follow-up evaluations). In our patients, we observed a quite preserved body composition, with alteration of the ratio between FM and FFM only in three patients. FFM mainly represents skeletal muscles, and it is chemically composed of water (extracellular: 29%; intracellular: 44%), proteins, glycogen, and minerals (Marra et al., 2019). Indeed, the myopathy in LOPD mainly involves proximal muscle, with preservation of leg and most arm muscles until later disease stages; thus, the evaluation of whole body FFM may not be able to detect myopathic changes until very late disease stages. Moreover, no patients had myopathy-related dysphagia and the hydration status was normal in all.

We know that high BMI signifies an overload for weak myopathic muscles and thus is expected to worsen motor and respiratory function. Moreover, we observed a negative effect of high BMI on motor outcome as assessed by the 6MWT. On the other hand, low BMI is also harmful since it is a poor prognostic index for myopathies and usually correlates with the worsening of respiratory function, together with increased risk of respiratory infections (Falagas et al., 2009). Decreased FFM as well as increased FM in patients with progressive degenerative diseases, including LOPD, may lead to an increased risk of adverse clinical outcomes, including greater mortality rates.

#### Correlations of FFM

We are here addressing a population with a disease that, we know, is expected to alter muscle structure and thus body composition. Thus, variations in body composition are better analyzed by comparison with disease parameters instead of by their absolute values. The correlation between FFM and creatine and 6MWT confirms that higher FFM reflects a better preserved muscle mass. Indeed, FFM correlated with baseline motor function, strength, and muscle mass by muscle MRI, too.

#### Phase Angle

This parameter, obtained by BIVA directly and not with regression equations, is thought to reflect the integrity of cell membranes (Marra et al., 2019). In several diseases (cancer, liver cirrhosis, renal failure, HIV infection and ALS) it has been regarded as a prognostic tool (Calcaterra et al., 2019; Garlini et al., 2019), reflecting critical ill disease. Phase Angle is independent of weight, BMI, age, and FFM/FM. This parameter has never been used before in the evaluation of a neuromuscular disorder.

We observed a relative stability of Phase Angle values in multiple measurements at different times in the same patients. The baseline value correlated with FFM (*p* = 0.025) and with BCM, which both represent the metabolically active mass. We noticed the utility of Phase Angle measurement in the follow-up, when we retrospectively noticed that patients with lower PhA at T0 showed a tendency to worsen over time. This data was confirmed by survival analysis. We do not know the reason why Phase Angle correlated with disease prognosis: patient 6 shows low PhA Z-scores and will experience severe motor worsening, thus possibly reflecting worse “muscle quality,” while a different situation occurs for patient 5, who had baseline muscle mass preserved and will experience severe respiratory worsening instead.

In our population, not including patients with critically ill disease nor dysphagia or major nutritional/hydration problems, it seems that low baseline PhA indicates patients that will develop more fragility (either motor or respiratory) and will need closer assessment and a stricter follow-up. We believe that Phase Angle reflects muscle status and muscle membrane integrity. Low Phase Angle may indicate less preserved skeletal muscle membrane integrity, for instance in case of increased muscle autophagic build-up, that is, known to affect negatively ERT response (Lim et al., 2018), and is considered as the main responsible for ERT resistance and ERT secondary decline even in responders. Indeed, ERT targeting requires membrane integrity. Unfortunately we did not search for correlations between low PhA and ERT pharmacokinetic profile/uptake, or possible biomarkers of autophagy (if available).

### Very Mild and Very Severe Disease: Criteria for Start and Stop

We have observed that patients with very severe disease also (*n* = 6), usually not included in clinical trials, may benefit from ERT (Supplementary Table S2). Indeed, also for patients who are wheelchair bound or ventilator dependent it is very important to retain their level of independence. It would therefore be useful to identify outcome measures specifically suited for patients with very severe disease, like functional scores for the upper limbs, other timed tests that can be completed even by severely motor impaired patients, the evaluation of the amount of hours without ventilation support, the walking speed analysis and so on. In a progressive myopathy like LOPD, even a slowed decline can be considered as a positive response: thus, we believe that, even in these cases, binding stop criteria should not be formulated, since every case should be discussed individually with the patient and family.

Our three asymptomatic patients remained clinically silent during the 15-years follow-up. Actually, one was mildly symptomatic, but the symptoms were judged by the patient himself as tolerable compared to the prospective of bimonthly infusions at the hospital. The use in all three patients of the same outcome measures we used for ERT monitoring shows that the course over time was stable, apart from the older patient in whom the mild decline in FVC and muscle function may be considered physiological for his age. Thus, there seems to be no reason to support starting ERT in pre-symptomatic patients. Our asymptomatic patients all had raised CK, which some Authors consider as an indicator of muscle suffering, and all had mild, non-specific muscle symptoms such as lumbar pain and fatigue, but no clinical signs. On the basis of our findings, we recommend monitoring of these patients over time, and once a year seems a sufficient time frame to detect timely any change in clinical status. Thus, we believe that treatment should not be started in the absence of skeletal muscle weakness (assessed by muscle strength tests or by 6MWT) and respiratory involvement (defined as FVC <80%). When specifically asked, all these patients report some myalgias, fatigue, or lumbar pain, but this is non-specific, and however never disabling, and vague symptoms do not seem to be a sufficient criterion for starting ERT, given the benign nature of their course of disease over time.

## Conclusion

Other studies have examined nutritional parameters in LOPD, but Phase Angle, considered a prognostic indicator for several diseases, was not assessed, neither in LOPD nor in other neuromuscular disorders. If our results will be supported by other studies, then BIVA could be useful in LOPD, not for investigating the nutritional status, but rather for assessing muscle membrane integrity and muscle quality. This could be especially important for predicting treatment response to a muscle-targeted treatment such as ERT.

Indeed, ERT targeting may be reduced in case of poor muscle membrane integrity, for instance in case of increased autophagy. Our study was undertaken in a small cohort and in an heterogeneous population as regards severity of involvement, and need thus confirmation by larger studies, on LOPD but ideally also on other myopathies. This evidence, if any, should be ideally supported by the assessment of biomarkers of muscle stress/ERT uptake/autophagy, as well as by clinical correlations with muscle strength or motor performances.

Although currently not easily available in neuromuscular settings, BIVA may allow the non–invasive follow-up of body composition modifications over time and, if our results will be confirmed, may be used as a marker of muscle health and quality, that may in turn be predictive of a poor response to ERT. This could help to individuate those patients in which a closer follow-up is required, due to higher fragility and risk of deterioration. If confirmed, this could mean that PhA may be a marker of muscle quality and BIVA may be a fast, easily performed and repeated, as well as cheap method, and can therefore serve as a valuable tool to predict ERT response in patients with LOPD, and to track changes over time and response to muscle targeted-interventions.

The best method for the evaluation of treatment response in LOPD is still under debate and the monitoring of disease progression has become more relevant since ERT availability in 2006, as also reflected by the dramatic increase of literature data on the search for proper outcome measures. This issue is not easily solved, since measures of muscle strength and function often require subjective collaboration, are penalized by scarce sensitivity to little changes, or are complicated by contemporary and reciprocal influence of more than one function: the patients’ performance on the 6MWT, for example, does not exclusively depend on motor function, but is also determined by the respiratory function. For the future, an assessment strategy as multi-comprehensive and multi-dimensional as possible would be desirable, possibly evaluating the same aspect of skeletal muscle with several tools in order to obtain confirmatory evaluations from different instrumental perspectives and to gain a more thorough understanding of the outcome.

## Data Availability

The datasets presented in this study can be found at Zenodo DOI: 10.521/zenodo.5814909.

## Footnotes

1
https://www.euro.who.int/en/health-topics/disease-prevention/nutrition/a-healthy-lifestyle/body-mass-index-bmi.
