# Supplementary material for: Bioimpedance Phase Angle as a Prognostic Tool in Late-Onset Pompe Disease: A Single-Centre Prospective Study With a 15-year Follow-Up
Source: Front Cell Dev Biol. 2022 Feb 18;10:793566. doi: 10.3389/fcell.2022.793566 (PMC8896115; doi:10.3389/fcell.2022.793566)
Supplement: Supplementary file 1 [file DataSheet1.docx]

Supplementary Material

# Supplementary Data

# Supplementary Tables

***Supplementary Table 1:*** *Demographic, clinical and genetic features of the patients followed-up at our center*

| **Patient, sex** | **ERT duration**  (y) | **Age at symptom onset** | **Age at ERT start**  **(disease duration at start ERT)** | **Actual age/age of death#** | **Walton score at the start of ERT** | **Respiratory support at the start of ERT** | **Walking support at the start of ERT** | **Second mutation*** | **Mutation impact / severity**** |
| --- | --- | --- | --- | --- | --- | --- | --- | --- | --- |
| 1, M | 13 | 21 | 28 (7) | 42 | 2 | no | no | 2104CT | potentially less severe |
| 2, M | 11 | 36 | 40 (4) | 51 | 1 | no | no | delexon18 | very severe |
| 3, M ° | 14 | 29 | 49 (29) | 63 | 3 | no | no | 525delT | very severe |
| 4, M | 14 | 42 | 54 (12) | 68 | 3 | no | no | 2298_2301delinsAAAGTA | very severe |
| 5, F | 11 | 32 | 39 (7) | 50 | 1 | no | no | 1694-1697delTCTC | very severe |
| 6, F | 13 | 7 | 28 (21) | 41 | 5 | no | monolateral support | 1297GA | less severe |
| 7, F | 14 | 47 | 60 (13) | 74 | 4 | NIV (nocturnal + 4 hours daily) started at 58 | no | 2530-41delEx18 | potentially less severe |
| 8, F | 8 | 30 | 39 (9) | 47 | 1 | no | no | 784GA | potentially less severe |
| 9, F | 9 | 60 | 72 (12) | 83# | 6 | no | walker | 784GA | potentially less severe |
| 10, F°° | 8 | 47 | 60 (13) | 68# | 3 | no | no | 525delT | very severe |
| 11, F | 9 | 48 | 58 (10) | 67 | 2.5 | NIV (nocturnal) started at 58 | no | 525delT | very severe |
| 12, F ° | 14 | 21 | 44 (23) | 58 | 2.5 | NIV (nocturnal) started at 41 | no | 525delT | very severe |
| 13, M | - | - | - | 34 | 0 | no | no | delTG2219-2220 | very severe |
| 14, M °° | - | 70 | - (12) | 82 | 2 | no | no | 525delT | very severe |
| 15, M | - | - | - | 41 | 0 | no | no | 525delT | very severe |
| 16, F | 4 | 40 | 61 (21) | 71# | 6 | invasive ventilation | walker < 40 mt | 2237 GA | very severe |
| 17, M | 5 | 24 | 52 (28) | 66 # | 6 | NIV (nocturnal + 8 hours daily) | walker | 1465 GA | potentially less severe |
| 18, M | 2 | 68 | 82 (14) | 84 # | 6 | NIV (nocturnal + 8 hours daily) + O_2_  2 L/min | walker <40 mt | 1076 G>C | very severe |
| 19, M | 15 | 24 | 49 (25) | 63 | 6 | invasive ventlation | wheelchair (not completely dependent) | 525delT | very severe |
| 20, F | 14 | 50 | 61 (11) | 76 | 7 | no | wheelchair | 1561G>A | potentially less severe |
| 21, F | 14 | 30 | 57 (27) | 71 | 7 | NIV (nocturnal 8 hours) started at 55 | wheelchair | 2237GA | very severe |

°, °°: pairs of siblings
*The first mutation is the IVS13T>G typically found in adults, for all patients

** GAA mutations were graded according to their presumed functional consequences on GAA activity, as less severe / potentially less severe / very severe, as described in the Pompe mutation database ([http://www.pompevariantdatabase.nl](http://www.pompevariantdatabase.nl/pompe_mutations_list.php?orderby=aMut_ID1)).

*Legend to Supplementary Table 1*. Patients 1 to 12 were on ERT; these patients were included in the longitudinal analysis of treatment effectiveness, whose inclusion criteria were: a) residual FVC > 30%; b) ability to complete the 6MWT test (ambulatory devices were allowed). Patients 14, 15 and 16 were not on ERT because either asymptomatic (patients 14 and 16) or with only mild symptoms and sign and thus deciding to refuse treatment (patient 15). Patients 17 to 22 had “severe” disease (defined as FVC <30%, invasive ventilation, inability to complete the 6MWT), implying limited reliability of motor and respiratory outcome measures, so they were excluded from the analysis of ERT effectiveness; this group included two patients requiring non invasive ventilation, one patient with FVC < 30%, and three patients who were either wheelchair-dependent or unable to complete the 6MWT.

***Supplementary Table 2:*** *Main clinical findings and outcome of patients with “very severe” disease*

| **Patient, sex** | **Age ERT start** | **Disease**  **duration at ERT start** | **Motor function at T0 (Walton score)** | **Respiratory function at T0**  **(daily hours of ventilation)** | **Outcome** |
| --- | --- | --- | --- | --- | --- |
| 16, F | 61 | 21 | Able to walk with aids (6) | Invasive ventilation 8 h/night + 8h/day | Motor and respiratory worsening, interrupts ERT at age 65, death at age 71 (respiratory infection) |
| 17, M | 52 | 28 | Able to walk with aids (6) | NIV 8 h/night  + 8 h/day | Motor and respiratory worsening, interrupts ERT at age 58, wheelchair at age 57, tracheotomy at age 60, death at age 66 (respiratory infection) |
| 18, M | 82 | 14 | Able to walk with aids (6) | NIV 8 h/night  + 8 h/day with  O_2_ 2 L/min | Respiratory worsening (concomitant cardiomyopathy), death after 2 years at age 84 due to cardiac failure |
| 19, M | 49 | 25 | Able to walk with aids (6) | Invasive ventilation 8 h/night + 4 h/day | Improved respiratory function, weight gain, able to walk a few meters without aid |
| 20, F | 61 | 11 | Wheelchair (7) | FVC 90% | Stable motor function and respiratory function |
| 21, F | 57 | 27 | Wheelchair (7) | NIV 8 h/night | Stable motor function and respiratory function |

*Legend to Supplementary Table 2* Six patients were either unable to walk or to complete the 6MWT (n=3), unable to collaborate in the execution of FVC measurement (n=1), or the evaluation of FVC measurement was unreliable due to tracheotomy (n=2). Thus, the evaluation of the main outcome measures was not possible or reliable. Three of these patients (patients 16, 17, 19) started ERT in 2005 through Expanded Access Programs (“compassionate use”), due to their severe clinical conditions (especially with regard to respiratory dysfunction, since it is the main life-threatening complication). The other three started ERT in the spring of 2006, as soon as marketing authorization of Myozyme became available. Patients 16 and 17 interrupted ERT after 4 and 6 years, respectively, due to objective worsening of their conditions: patient 16 had increasing trouble with ambulation (he became completely wheelchair dependent 1 year after ERT cessation) and had recurrent respiratory infections; patient 17 became wheelchair dependent and his respiratory function also worsened while on ERT, until he required a tracheotomy during a respiratory infection. Both patients had also subjectively perceived the lack of any clinical improvement, so that interruption of the treatment was decided by the patients themselves. They survived for another 6 and 8 years after ERT discontinuation, although in severe clinical conditions, meaning that they were on invasive ventilation 24/24h, wheelchair bound, and required a percutaneous gastrostomy for alimentation. Both died for respiratory infections.

Patient 18 was 82 when started ERT: he was probably one of the oldest patients that ever started ERT, at least in Italy. He had severe cardiomyopathy, possibly secondary to the severe respiratory dysfunction. His condition remained stable in its severity during the first two years of treatment, then he died due to pulmonary edema secondary to cardiac failure.

Patient 19, when he started ERT, was both tracheotomized and wheelchair bound (even though he was not completely dependent on the use of the wheelchair), with signs of severe malnutrition. He was the first adult patient to be treated in Italy, through Expanded Access Programs, in view of his severe condition. During the first year of ERT he gained 13 kg that represented 30% of his bodyweight, and improved respiratory function (reduced daily hours of ventilation). This respiratory improvement progressed over the following two to three years and then stabilized and has remained stable since then.

Patient 20 and 21 are still wheelchair bound, and their respiratory function remains quite stable after 15 years of follow-up.

***Supplementary Table 3:*** *Baseline motor and respiratory function of the patients at ERT start*

| **Pt, sex, motor/ respiratory function at baseline** | **ERT start** (year) | **MWT T0** % | **MWT T0** (meters) | **FVC upright**  **T0** % | **FVC upright  T0** (liters) | **Outcome/event during ERT and timing** (year) |
| --- | --- | --- | --- | --- | --- | --- |
| 1, M | 2007 | 78 | 495 | 85 | 4.26 | *T9 |
| 2, M | 2009 | 90 | 420 | 78 | 3.68 |  |
| 3, M | 2007 | 65 | 394 | 92 | 4.18 |  |
| 4, M | 2007 | 69 | 480 | 60 | 2.80 | #T9 |
| 5, F | 2009 | 88 | 360 | 82 | 2.45 | *T6 |
| 6, F # | 2007 | 32 | 214 | 78 | 2.83 | ## T5 |
| 7, F * | 2006 | 55 | 256 | 42 | 0.83 |  |
| 8, F | 2012 | 96 | 580 | 98 | 3 |  |
| 9, F # | 2007 | 30 | 60 | 68 | 1.6 | ## T4  Death due to sepsis at T11 |
| 10 F | 2006 | 52 | 256 | 67 | 1.47 | *T2, #T7  Death due to metastatic breast cancer at T8 |
| 11, F * | 2011 | 99 | 513 | 100 | 2.9 |  |
| 12, F * | 2006 | 62 | 384 | 30 | 0.9 |  |
| 16 F **, # | 2005 | Unable to complete | 18 | invasive ventilation | 0.7 (29%) | ## T5,  ERT interrupted after 4 years, death at T10 due to respiratory failure |
| 17, M *, # | 2005 | <20% | 75 | <30% | 0.9 (28%) | ## T5, ** T8  ERT interrupted after 6 years, death at T14 due to respiratory failure |
| 18, M *, # | 2006 | <10% | 22 | <30% | Unable to perform | ERT for 2 years, death at T2 due to cardiac failure |
| 19, M **, ## | 2005 | Unable to complete | - | Invasive ventilation | 0.67 (16%) | Improvement in respiratory function, then stabilization |
| 20, F ## | 2006 | Unable to complete | - |  | 2.4 (90%) | Stable |
| 21, F *, ## | 2006 | Unable to complete | - |  | 1.22 (51%) | Stable |

Requirement for *NIV or **invasive ventilation (tracheostomy) at the time of ERT start (first column) or during ERT (last column); requirement for #walking aid or ##wheelchair at the time of ERT start (first column) or during ERT (last column)

Patients 16 to 21 are those labelled as “very severe” disease

*Legend to Supplementary Table 3* Baseline 6MWT and FVC are expressed both as absolute values and as % compared to normal controls matched for age, sex, BMI). The last column illustrates the patients in whom a major change in motor functional status (requirement of walking aids) or respiratory status (requirement of ventilator support) occurred during ERT, while the first column illustrates patients that already relied on walking aids (#, ##) or respiratory support (*, **) at the time of ERT start.

Of the 12 patients included in the outcome analysis (patients 1 to 12), patients 8 and 11 had normal FVC (defined as FVC > 80%) and normal 6MWT, but were started on ERT because of reduced muscle strength of lower limbs (thigh adductors, hip extensors and knee flexors) and postural changes (anserine gait); three patients (patients 7, 11, 12) were on nocturnal non-invasive ventilation; all but two patients (patients 6 and 9) were able to walk unassisted; two patients died during the follow-up: a) patient 9 became wheelchair dependent during ERT and respiratory function also worsened, she developed dysphagia and severe weight loss but refused enteral or parenteral nutritional support, the she died due to systemic infection, possibly as a consequence of protein and caloric malnutrition, b) patient 10 died 8 years after ERT start for complications of metastatic breast cancer, with lung metastasis that possibly worsened the already severe respiratory dysfunction. During the previous 8 year she had shown a poor response to ERT, with the need of nocturnal ventilatory support after 2 years of ERT, and the need of walking devices after 7 years of ERT.

***Supplementary Table 4:*** *Summary of 6MWT over time*

The table includes T0-T1 and then time points every three years, with calculation of the absolute and percentage variations between T0 and T1 and between T6 and T0 and then between the final follow-up and T0. An increase >10% or >30m is labelled as improvement (**↑)**, between -10 and +10% (or between –30 and +30m) as stabilization (=); decrease < -10% or < -30m is labelled as worsening (**↓)**.

| **Pt** | **ERT start** (year) | **MWT  T0**  (m) | **MWT  T1**  (m) | **MWT**  **T1-T0**  (m, %) | **MWT  T3** (m) | **MWT  T6** (m) | **MWT**  **T6-T0**  (m, %) | **MWT  T8/9** (m) | **MWT  T11/12** (m) | **Final MWT** (m, year) | **MWT**  **Tfinal-T0** (m, %) | **Syn-thesis*** |
| --- | --- | --- | --- | --- | --- | --- | --- | --- | --- | --- | --- | --- |
| 1 | 2007 | 495 | 576 | 81 (16.36%) **↑** | 528 | 492 | -3 (-0,61%) **=** | 490 | 490 | 490 (T13) | -5 (-1.01%) **=** | 2 |
| 2 | 2009 | 420 | 597 | 177 (42.14%) **↑** | 486 | 480 | 60 (14.29%) **↑** | 450 | 436 | 436 (T11) | 16 (3.81%) **=** | 1 |
| 3 | 2007 | 394 | 416 | 22 (5.58%) **=** | 440 | 498 | 104 (26.4%) **↑** | 462 | 438 | 417 (T14) | 23 (5.84%) **=** | 1 |
| 4 | 2007 | 480 | 512 | 32 (6.67%) **=** | 426 | 360 | -120 (-25%) **↓** | 267 | 250 | 222 (T14) | -258 (-53.75%) **↓** | 5 |
| 5 | 2009 | 360 | 510 | 150 (41.67%) **↑** | 366 | 426 | 66 (18.33%) **↑** | 364 | 300 | 300 (T11) | -60 (-16.67%) **↓** | 3 |
| 6 | 2007 | 214 | 246 | 32 (14.95%) **↑** | 150 | 0 | -214 (-100%) **↓** | 0 | 0 | 0 (T13) | -214 (-100%) **↓** | 3 |
| 7 | 2006 | 256 | 352 | 96 (37.5%) **↑** | 384 | 324 | 68 (26.56%) **↑** | 333 | 318 | 316 (T14) | 60 (23.44%) **↑** | 1 |
| 8 | 2012 | 580 | 598 | 18 (3.1%) = | 580 | 555 | -25 (-4.31%) **=** | 590 | - | 590 (T8) | 10 (1.72%) **=** | 4 |
| 9 | 2007 | 60 | 64 | 4 (6.67%) **=** | 58 | 25 | -35 (-58.33%) **↓** | 10 | - | 0 (T11) | -60 (-100%) **↓** | 5 |
| 10 | 2006 | 256 | 321 | 65 (25.39%) **↑** | 315 | 252 | -4 (-1.56%) **=** | 136 | - | 136 (T8) | -120 (-46.88%) **↓** | 3 |
| 11 | 2011 | 513 | 456 | -57 (-11.11%) **↓** | 405 | 336 | -177 (-34.5%) **↓** | 460 | - | 448 (T9) | -65 (-12.67%) **↓** | 6 |
| 12 | 2006 | 384 | 450 | 66 (17.19%) **↑** | 389 | 460 | 76 (19.79%) **↑** | 399 | 360 | 359 (T14) | -25 (-6.51%) **=** | 2 |

*Synthesis of 6MWT course over time according to a scoring system proposed by Harlaar et al. (with grading from best to worst outcome): 1) improvement throughout the study; 2) initial improvement followed by stabilization; 3) initial improvement followed by decline; 4) stable over time; 5) initial stabilization then decline; 6) initial decline then stabilization; 7) decline throughout the study; 8) unclassifiable (Harlaar et al. 2019).

Patient 3 is classified as 1 since, while at T1 the magnitude of the improvement is not as high as 10% or 30m, the global course is of constant improvement.

Patient 5 is classified as 3 for improved values at T0 and T6, but we see a tardive and consistent decline at the final follow up, not attributable to other conditions nor to aging, so we classify her as initial improvement followed by decline = 3 (we see that decline may be tardive, after the initial 6 years of improvement).

Patient 8 is labelled as 4 since 6MWT did not change over time, but it is a normal 6MWT, so we see the limits of this grading system to consider a bad outcome a sum of respiratory + motor value > 8.

Patient 11 is labelled as decline throughout the study since values are always lower compared to T0, but however rather stable on time and with good motor performances.

Thus, we see the limits of this grading system that labels two patients with normal 6MWT (patients 8 and 11), who are relatively stable over time, with high scores (4 and 6).

*Legend to Supplementary Table 3*  The overall improvement is evident during the first year (p=0.010), with a tendency to return to T0 values within the first three years, followed by a relative stabilization and then a slow worsening, beginning 6 years after ERT start and continuing thereafter (p=0.04 after T9 and p=0.026 after T12)

At the final follow-up, the 6MWT is usually lower than at the start of treatment (mean -15.9 + 35.64 %, range +23 to -100 %).

Individual variations at T1 range -11% to +42% (and from -57 to +177 meters) compared to the baseline, with 7 patients with improvement > 10% or > 30 meters, one patient with worsening < -10% or < -30 meters, and 4 patients stable (variations between -10 and +10% or between -30 and +30 meters). After 6 years, variations compared to T0 ranged from -100% to +26%, with 5 patients still showing improvement > 10% compared to the baseline, 4 patients with worsening < -10% and the remaining three patients stable. The final motor outcome (8 to 14 years follow-up) is usually stable compared to the 6-year outcome, but exceptions are found with tardive deteriorations (patient 5 for instance).

***Supplementary Table 5:*** *Summary of FVC over time*

The table includes T0-T1 and then time points every three years, with calculation of the absolute and percentage variations between T0 and T1 and between T6 and T0 and then between the final follow-up and T0. A variation > 10% or ≥ 200 ml is considered as the minimal clinically important change of improvement (↑), worsening (↓) is defined as a change < -10% or < -200 ml, and the remainders are considered stable (=).

| **Pt** | **ERT start** (year) | **FVC**  **T0**  (L) | **FVC**  **T1**  (L) | **FVC**  **T0-T1**  (Liters, %) | **FVC**  **T3**  (L) | **FVC**  **T6**  (L) | **FVC**  **T6-T0**  (Liters, %) | **FVC**  **T8/9**  (L) | **FVC**  **T11/12**  (L) | **Final FVC**  (L, year) | **FVC**  **Tfinal-T0**  (Liters, %) | **Syn-thesis*** |
| --- | --- | --- | --- | --- | --- | --- | --- | --- | --- | --- | --- | --- |
| 1 | 2007 | 4.26 | 4.09 | -0.17 (-3.99%) **=** | 3.9 | 3.4 | -0.86 (-20.19%) **↓** | 3.2 | 3.5 | 3.6 (T13) | -0.66 (-15.49%) **↓** | 5 |
| 2 | 2009 | 3.68 | 4.09 | 0.41 (11.14%) **↑** | 4.3 | 3.91 | 0.23 (6.25%)  **=** | 3.1 | 3 | 3.08 (T11) | -0.6 (-16.3%) **↓** | 2 |
| 3 | 2007 | 4.18 | 4.22 | 0.04 (0.96%) **=** | 3.9 | 4.25 | 0.07 (1.67%)  **=** | 3.55 | 2.73 | 3.68 (T14) | -0.5 (-11.96%) **↓** | 4 |
| 4 | 2007 | 2.8 | 2.38 | -0.42 (-15%) **↓** | 1.92 | 2.03 | -0.77 (-27.5%) **↓** | 1.71 | 2.01 | 2.01 (T14) | -0.79 (-28.21%) **↓** | 7 |
| 5 | 2009 | 2.45 | 2.59 | 0.14 (5.71%) **=** | 2.3 | 1.65 | -0.8 (-32.65%) **↓** | 1 | 0.9 | 0.9 (T11) | -1.55 (-63.27%) **↓** | 5 |
| 6 | 2007 | 2.83 | 2.9 | 0.07 (2.47%) **=** | 2.7 | 2.6 | -0.23 (-8.13%) **=** | 2.9 | 2.6 | 2.6 (T13) | -0.23 (-8.13%) **=** | 4 |
| 7 | 2006 | 0.83 | 1.05 | 0.22 (26.51%) **↑** | 1.02 | 1.07 | 0.24 (28.92%) **↑** | 0.91 | 1.03 | 0.93 (T14) | 0.1 (12.05%) **=** | 2 |
| 8 | 2012 | 3 | 3.33 | 0.33 (11%) **↑** | 3.31 | 3.61 | 0.61 (20.33%) **↑** | 3.7 | - | 3.7 (T8) | 0.7 (23.33%) **↑** | 1 |
| 9 | 2007 | 1.6 | 1.5 | -0.1 (-6.25%) **=** | 1.29 | 1.42 | -0.18 (-11.25%) **↓** | 1.19 | - | 1.19 (T11) | -0.41 (-25.63%) **↓** | 5 |
| 10 | 2006 | 1.47 | 1.18 | -0.29 (-19.73%) **↓** | 2.19 | 2.12 | 0.65 (44.22%) **↑** | 1.37 | - | 1.37 (T8) | -0.10 (-6.8%) **=** | 6 |
| 11 | 2011 | 2.9 | 2.77 | -0.13 (-4.48%) **=** | 2.89 | 2.61 | -0.29 (-10%) **↓** | - | 2.6 | 2.61 (T9) | -0.29 (-10%) **↓** | 5 |
| 12 | 2006 | 0.9 | 0.93 | 0.03 (3.33%) **=** | 1.06 | 1.04 | 0.14 (15.56%) **↑** | 0.66 | 0.85 | 0.83 (T14) | -0.07 (-7.78%) **=** | 4 |

*Synthesis of FVC course over time according to a scoring system proposed by Harlaar et al. (with grading from best to worst outcome): 1) improvement throughout the study; 2) initial improvement followed by stabilization; 3) initial improvement followed by decline; 4) stable over time; 5) initial stabilization then decline; 6) initial decline then stabilization; 7) decline throughout the study; 8) unclassifiable (Harlaar et al. 2019).

Patient 2 is classified as 2 despite later decline, since the decline is very tardive and very mild (only -16.3% compared to baseline).

Patient 3 is classified as 4 corresponding to “stable” since the decline is very late and very mild (-11.96%), also considering that at the last follow-up his age was >60.

*Legend to Supplementary Table 5* the changes in FVC over time are shown: we considered a variation > 10% from baseline or ≥ 200 ml as the minimal clinically important change that defines improvement or worsening beyond the measurement error.

***Supplementary Table 6:*** *Comparison of different scoring methods to assess the overall outcome and response to treatment*

|  | **(A)** | | | **(B)** | **(C)** |
| --- | --- | --- | --- | --- | --- |
|  | **Harlaar et al. scoring system*** | | | **Sum of final FVC+6MWT % variations as compared to baseline** | **Major events  during ERT** (year) |
| **Pt** | **FVC** | **MWT** | **FVC +  6MWT** |  |  |
| 1 | 5 | 2 | 7 | -16% | NIV (T9) |
| 2 | 2 | 1 | 3 | -14% |  |
| 3 | 4 | 1 | 5 | -5% |  |
| 4 | 7 | 5 | 12 | -80% | Walking aid (T9) |
| 5 | 5 | 3 | 8 | -85% | NIV (T6) |
| 6 | 4 | 3 | 7 | -108% | Wheelchair (T5) |
| 7 | 2 | 1 | 3 | 35% |  |
| 8 | 1 | 4 | 5 | 20% |  |
| 9 | 5 | 5 | 10 | -125% | Wheelchair (T4) |
| 10 | 6 | 3 | 9 | -47% | NIV (T2), wheelchair (T7) |
| 11 | 5 | 6 | 11 | -22% |  |
| 12 | 4 | 2 | 6 | 2% |  |

*The scores of the Harlaar et al. scoring system has the merit of attempting to provide a synthesis of the FVC and 6MWT outcome course over time (with grading from best to worst outcome: 1) improvement throughout the study; 2) initial improvement followed by stabilization; 3) initial improvement followed by decline; 4) stable over time; 5) initial stabilization then decline; 6) initial decline then stabilization; 7) decline throughout the study; 8) unclassifiable), as well as a sum score of the respiratory and motor outcome, with lower scores corresponding to a better overall outcome and higher scores to a worse overall outcome (Harlaar et al. 2019, ref 18).

*Legend to Supplementary Table 6.* The table compares three different scoring methods to assess the overall outcome: (A) the scoring system proposed by Harlaar et al.; (B) the sum of % changes in the final 6MWT + FVC; and (C) the occurrence of “major events”, defined as the need for walking or ventilatory support, or major changes in walking abilities/respiratory functions/needs for support /hours of ventilation, occurring during ERT.

The table illustrates the limitations of the Harlaar et al. scoring system (A): for example, patient 6, who loses her ability to walk but has preserved respiration, scores better than the rather stable patient 11, in whom 6MWT and FVC both worsen, but without impacting her functional abilities through the entire follow-up.

The sum of percentage variation scoring system (B) also has limitations: we see that patient 1, who is young and with mild disease stage at inclusion (so that we expect a good response), and developing severe respiratory decline until requirement of NIV, scores better than patient 4, who has marked reduction of the final percentages but no real functional change (he was 54 when starting ERT and with already severe baseline involvement, and he is now 67 and able to walk unassisted and without ventilatory support, thus we think it is correct to consider him as a good responder).

In view of these limitations, we decided to adopt the occurrence of “major events” (C) to assess overall outcome and response to treatment and perform survival analysis, which has the additional merit to take into account the timing of the event, too. We thus identified the patients 1, 5, 6, 9, and 10 as non-responders.

***Supplementary Table 7:*** *Summary of nutritional parameters of the 12 patients*

|  | | **BMI  T0** | **TBW/ weight** | **ECW/ TBW** | **FM%  T0** | **FFM%  T0** | **BCM%/ FFM** | **BCM variation from normal** | **Z-score Phase Angle** |
| --- | --- | --- | --- | --- | --- | --- | --- | --- | --- |
| Mean | | 24.5408 | -0.3182 | 5.4182 | 27.42 | 73.1 | 48.591 | 7.9417 | -1.0167 |
| Median | | 23.125 | 1.2 | 4.3 | 26.65 | 73.35 | 48.7 | 7.4 | -0.8 |
| Standard deviation | | 6.40331 | 5.6593 | 4.72923 | 10.1 | 10.8599 | 5.4438 | 4.46389 | 0.69653 |
| Percentiles | 25 | 21.6 | -4 | 1.7 | 22.525 | 67.2 | 45 | 5.025 | -1.725 |
|  | 50 | 23.125 | 1.2 | 4.3 | 26.65 | 73.35 | 48.7 | 7.4 | -0.8 |
|  | 75 | 26.5125 | 3.6 | 9.8 | 32.8 | 78.975 | 54 | 11.225 | -0.425 |

TBW= total body water

ECW = extracellular water (metabolically inactive, composed mainly of sodium)

ICW = intracellular water

TBW/weight = percentage of the weight that is represented by water

BCM = metabolically active tissue, composed mainly of ICW and main electrolyte is potassium

Indices of better “health” are: higher PhA, higher FFM, higher BCM, lower ECW, lower FM, lower ECM/BCM, lower BMI, lower ECW/TBW, higher ICW, lower ECW, higher ICW/TBW ratio

PhA is measured directly by the impedentiometer; FM, FFM and BCM are derived from regression equations

***Supplementary Table 8:*** *Nutritional parameters and outcomes in the 12 patients on ERT and in the three asymptomatic patients*

| **Patient,** (*Major Event) | **BMI  T0** | **FM%  T0** | **FFM% T0** | **Phase Angle  Z- score** | **Phase Angle °** | **PhA  at the last follow-up** | **FVC  T0-Tlast%** | **MWT  T0-Tlast%** |
| --- | --- | --- | --- | --- | --- | --- | --- | --- |
| 1* | 23.05 | 19.6 | 80.4 | -1.1 | 6.1 | -0.4 | -15.49 | -1.01 |
| 2 | 25.65 | 22 | 80 | -0.6 | 6.6 | -0.7 | -16.3 | 3.81 |
| 3 | 19.93 | 12.7 | 90.6 | -1.5 | 5.1 | -1.4 | -11.96 | 5.84 |
| 4 | 29.41 | 27 | 73 | -0.5 | 5.6 | -1 | -28.21 | -53.75 |
| 5* | 21.4 | 26.3 | 73.7 | -2 | 4.5 | -0.1 | -63.72 | -16.67 |
| 6* | 41 | 50.5 | 49.5 | -2.2 | 4.5 | -0.6 | -8.13 | -100 |
| 7 | 23.2 | 29.2 | 70.8 | -0.4 | 5 | -0.3 | 12.05 | 23.44 |
| 8 | 22.96 | 28.3 | 71.7 | -0.3 | 5.1 | 0.5 | 23.33 | 1.72 |
| 9* | 22.2 | 25.3 | 74.7 | -0.3 | 4.3 | -0.6 | -25.63 | -100 |
| 10* | 24.69 | 36.7 | 63.3 | -1.8 | 3.6 | -0.3 | -6.8 | -46.88 |
| 11 | 26.8 | 34 | 66 | -0.5 | 4.9 | -0.3 | -10 | -12.67 |
| 12 | 14.2 | 13 | 88 | -1 | 4.8 | -0.6 | 7.78 | -6.51 |
| 13 | 18.7 | 8.4 | 91.6 | -0.9 | 6.6 | 0.3 | 21.21 | 37.32 |
| 14 | 25.9 | 22.7 | 77.3 | -0.6 | 4.7 | -0.2 | -22 | -24 |
| 15 | 17.12 | 8.2 | 91.8 | -0.3 | 6.9 | -0.6 | 3 | 21 |

The most severe alteration in body composition is observed in patient 6, showing high BMI, high FM and low FFM (“sarcopenic obesity”).

Patient 10 shows normal BMI but a disproportion between FM (increased) and FFM (decreased).

Patient 4 show high BMI but a body composition that is quite normal (only mild increase in FM).

We can observe low PhA Z-score in patients with apparently normal body composition, such as patient 5, in which a severe respiratory worsening occurred during the follow-up.

## Supplementary Figures

***Supplementary Figure 1:*** *Disease course of individual patients over time and main disease related events*


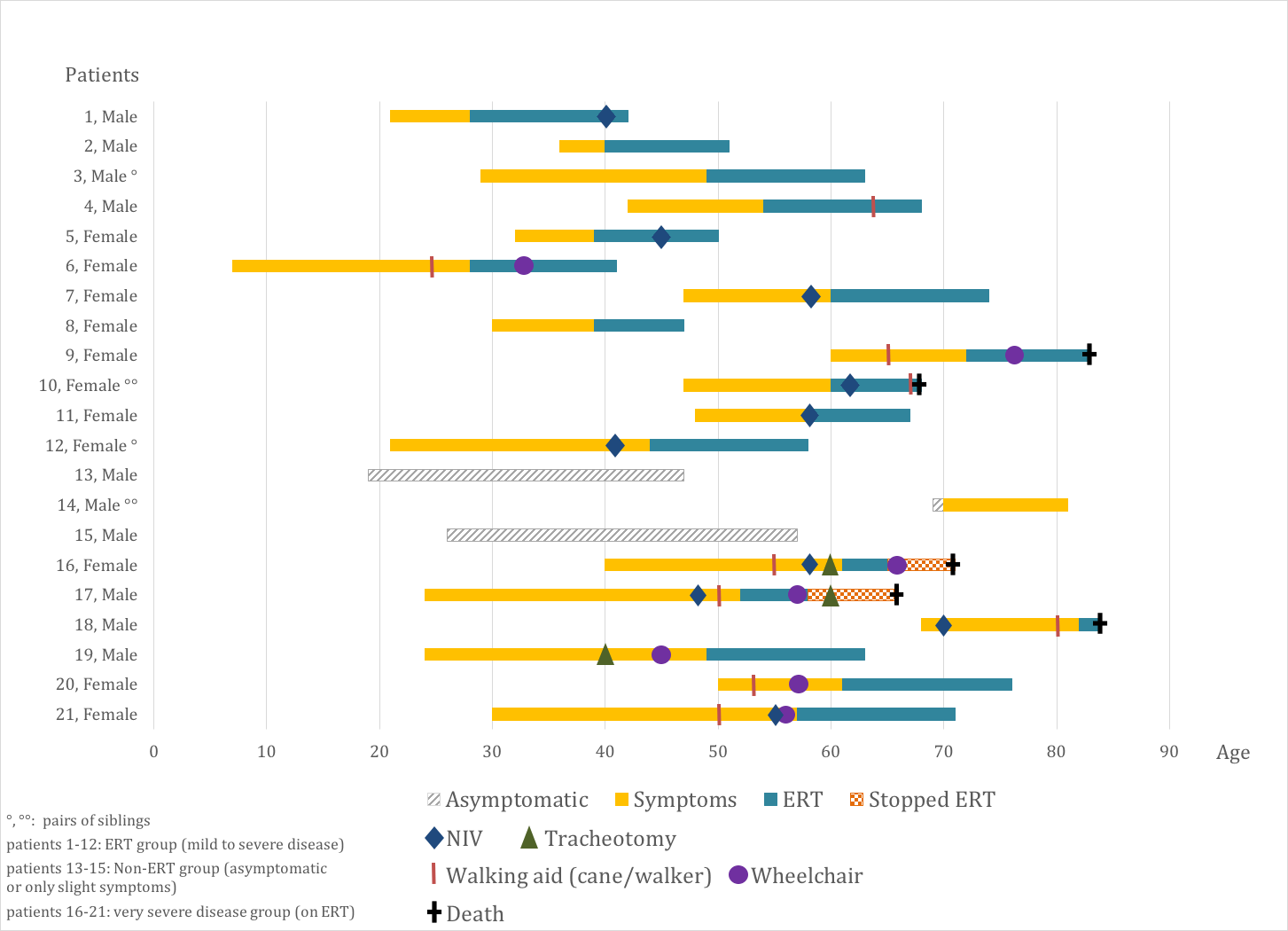


Each bar represents the course of disease of one individual patient. The length of each bar represents the duration of diagnosed, but asymptomatic disease (cross-hatched grey), the duration of symptomatic disease before the start of enzyme replacement therapy (ERT) (yellow), the duration of the follow-up during ERT (blue), and the follow-up duration after stopping ERT (chequered orange). The age at the start of using non-invasive ventilation (dark blue diamond), invasive ventilation (dark green triangle), a walking aid (red line) or a wheelchair (purple circle), and the age of death (black cross), is also indicated.

***Legend to Supplementary Figure 1*** Patient 13 and patient 15, followed-up at our center since 2005, never developed signs of LOPD during a 15-year follow-up. They were both diagnosed by muscle biopsy, which had been performed due to persistently raised CK, at age 19 (patient 13) and 26 (patient 15). Patient 14 was diagnosed at 70 in the context of a protocol for the screening of asymptomatic family members: he is the brother of patient 10, who had shown a more severe disease course (first symptoms and signs at 47; she was 60 when she started ERT and, at this time, she had moderate motor and respiratory involvement). At the time of diagnosis, patient 14 had mild postural changes (anserine gait with mild hyperlordosis) and mild difficulty in raising stairs. He had also noticed these changes but had attributed them to orthopedic problems or physiological aging, and did not have them investigated further, despite knowing of the genetic disease of his sister. All three patients have been followed–up yearly for 12 to 15 years. When specifically asked, all patients had some muscle symptoms, but in the form of vague and non-specific symptoms such as lumbar pain, fatigue or asthenia. Patients 13 and 15 never developed muscle weakness or postural changes. Only the older patient (patient 14) showed progression of motor and respiratory dysfunction, however not impacting his quality of life: indeed, he always refused to start ERT since he judged his deficits as acceptable compared to the prospective of bimonthly ERT infusions at the hospital.

**Supplementary Figure 2 ABCDE – secondary outcome measures**

**Supplementary Figure 2A** *Muscle strength of anterior, medial and posterior thigh muscles as measured by Hand Held Dinamometry at different time points* during the follow-up period

Ant = anterior thigh muscles, knee extensors (at T0, T1 and last follow-up)
Med = medial thigh muscles, thigh adductors (at T0, T1 and last follow-up)
Post = posterior thigh muscles, knee flexors (at T0, T1 and last follow-up)

| - - 1. Muscle strength (Newton) | - - 1. 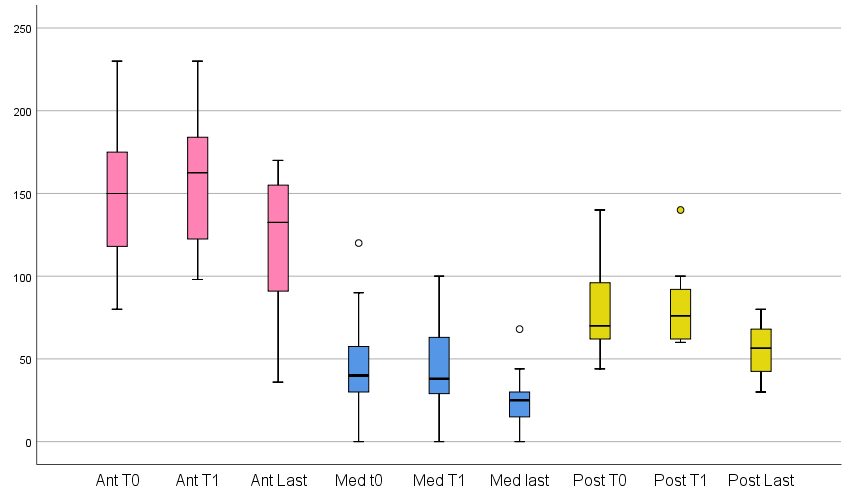 |
| --- | --- |

An initial, small improvement of muscle strength occurred only in the anterior thigh (155.6+43.8 Newton at baseline vs. 157.6+41.7 Newton at T1, p=0.05), while the medial and posterior thigh muscle strength remained stable during the first year. This was followed by a secondary decline in muscle strength for all thigh segments, with a more evident decline for the posterior and medial segments. The adductor muscles of the medial thigh were compromised the most (anterior thigh 155.6+43.8 Newton at baseline vs. 123.6+42.6 Newton at last follow-up, p=0.005; medial thigh 60.8+45.6 vs. 26.3+17.3 Newton, p=0.032; posterior thigh 93.2+43 vs. 55.9+16.3 Newton, p=0.05).

**Supplementary Figure 2B**

*Correlation between thigh muscle involvement on MRI and muscle strength.*

Mean MRI Mercuri score worsened of various degrees in all patients, with % decrease that were more pronounced in the posterior thigh (anterior thigh 13.5+6.3 vs. 18.8+ 10.6, p=0.012; medial thigh 16.9+5.9 vs. 20.9+9.5 p=0.008; posterior thigh 18.9+5.3 vs. 24.7+9.2 p=0.003). Mercuri score correlated with 6MWT, especially the anterior thigh score (rho -0.7, p=0.007 for anterior thigh, rho=-0.67, p=0.011 for medial thigh, rho -0.62, p=0.024 for posterior thigh), that is the segment including quadriceps femoris, which is more important for walking abilities. Muscle MRI generally correlated with muscle strength findings at the same segments, here the anterior and medial thigh are shown (R^2^ is 0.3, 0.6 respectively).

**Supplementary Figure 2C:**

Individual patients’ maximal inspiratory & expiratory pressures (MIP & MEP)

The blue bars represent the baseline value, the green bars are within 3-year follow-up and generally show an improvement, while the red bars are the last follow-up, usually T6-T9, and generally show values similar to the baseline. The patient numbers correspond to their identity as described in the other tables.

Mean MIP at T0 was -46.8 + 29 cm H_2_O, with a non-significant trend towards improvement at T3 (-51.5 + 25 cm H_2_O, with high variability in different patients) and a significant worsening at T6 (-37 + 17 cm H_2_O, p=0.028) compared to T3 values, followed by a milder, slower, non-significant decline over time (T12: -32+18 cm H_2_O). As expected, MIP values correlate with FVC.

Mean MEP value at baseline was 68 + 23 cm H_2_O, with a non-significant variation at T3 (72.6+25 cm H_2_O), a tendency to decline at T6 (61.6+36 cm H_2_O) and with final values (T9-T12: 47.4 + 17 cm H_2_O) lower than at baseline; however, all variations were non-significant, probably in view of the high variability of MEP in different patients and at different time points, possibly due to non-optimal collaboration. Values correlate with FVC. Patients 6 only shows a discrepancy between FVC (preserved) and MEP (weak) possibly due to severe obesity and inability to contract successfully the abdominal muscles, contributing to forced expiration.

Supplementary Figure 2D

*Changes in upright FVC vs. supine FVC*


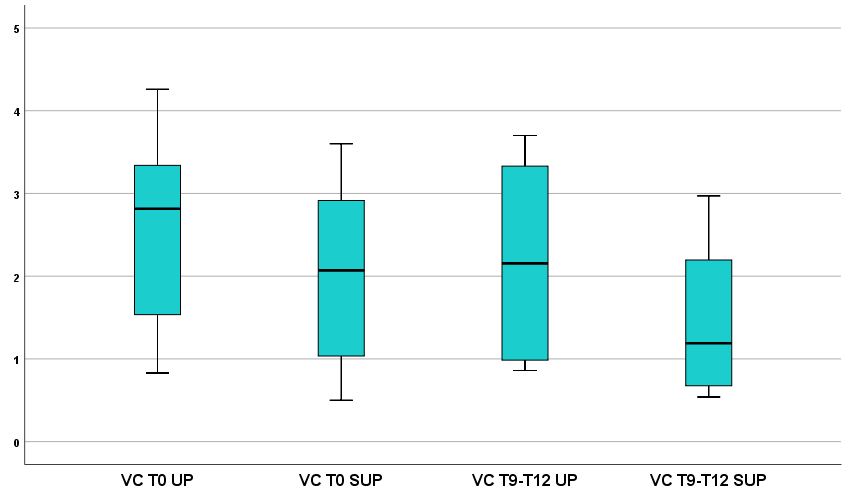


The box plots show that while upright FVC values tend to remain relatively stable during follow-up (slight decline, p=0.022), supine FVC values undergo more pronounced worsening in the same period of time (p=0.004).

While upright VC decreases from a median of 2.5 + 1.17 to 2.15 + 1.17 (p=0.022 ), supine VC decreased more consistently over the same time period (8-12 years): 2.04 + 1.05 at T0 vs 1.44 + 0.9 at the last follow-up (p=0.004) and postural drop changed from 22 + 9.4% at T0 to 33.5 + 10.5 at the last follow-up (p=0.003).

***Supplementary Figure 2E:***  *Postural drop at last follow-up as compared to baseline postural drop in individual patients*

1. The blue bars indicate the individual patients’ postural drop value at inclusion, the red bars indicate the postural drop value measured at the last follow-up. Values ≥20% postural drop (above the horizontal red line) indicate diaphragmatic weakness. It can be seen that while at inclusion only 6 patients had a postural drop ≥20%, at the last follow-up 12 patients had a postural drop ≥20%.
2. While at baseline 40% of patients had a postural drop ≥20%, at the last follow-up it was 80% of patients.

A


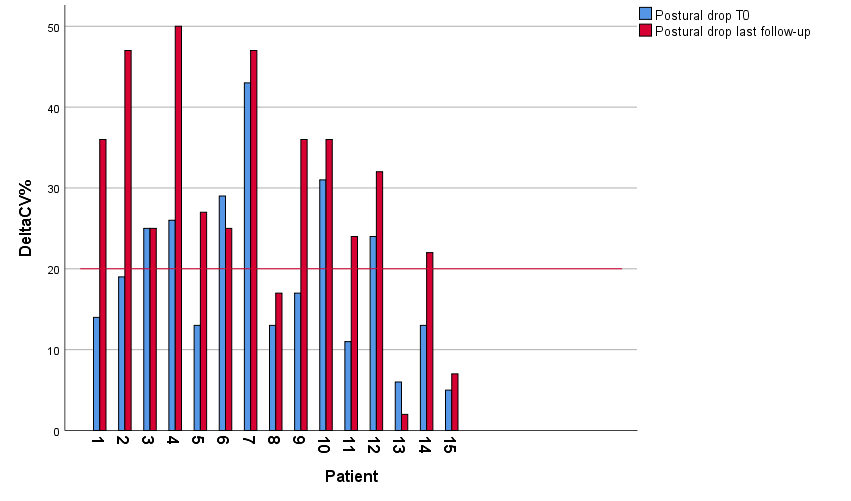


B

***Supplementary Figure 3A*** *Proportion of patients with either improvement, stabilization or decline of the 6MWT at three time points: T1, T6, and last follow-up (T8-T14).*

***Figure 3B:*** *Proportion of patients with either improvement, stabilization or decline of FVC at three time points: T1, T6, and last follow-up (T8-T14).*

***Figure 3C:***  *Summary of combined motor (6MWT) and respiratory (FVC) outcome over time (at T3, T6, and last follow-up, compared to T0).*

*Legend to Figure 3* Clinically meaningful changes are defined as: variations of >10% or >30 meters for 6MWT, variations >10% or >200 ml for FVC, as compared to baseline values. *Green*: improvement/stabilization in both outcome measures (6MWT and FVC); *Blue:* improvement/stabilization in one of the two outcome measures (6MWT or FVC); *Red:* worsening in both outcome measures (6MWT and FVC). The numbers inside the pie charts indicate the number of patients (n). Figure 3C summarizes the combined respiratory and motor outcome over time, by indicating the proportion of patients with (a) both outcome measures (6MWT and FVC) improved/stable, (b) either one of the outcome measures improved/stable or (c) both outcome measures worse (as compared to baseline values) at different time points during the follow up (T3, T6, and last follow-up).

The disease course shows high inter-individual variability, with some patients improving during the whole follow-up, others with initial improvement followed by stabilization or even worsening, and others initially stable and then declining, or initially declining and then stable

***Supplementary Figure 4:*** *Correlation between PhA Z-score, FM, FFM, initial and follow-up MRI in each patient*

Initial PhA Z-scores vary between -0.3 and -2.2, independent of the severity of fatty muscle degeneration (i.e. patient 5 has mild muscle involvement on MRI, but unexpectedly low PhA). During a 8-12 years follow-up there is variable progression of MRI fatty degeneration in all patients, from very mild to severe. Some patients with lower PhA Z-score show marked progression of MRI fatty degeneration (i.e. patients 6 and 10), while others (i.e. patient 5) show rather stable muscle MRI, but we know that she had severe respiratory progression. Therefore, PhA may be an indicator that can alert us to particularly fragile patients, who are at high risk for experiencing any type of clinical worsening.

| **Pt** | **Z-score Phase angle** | **FM%* T0**  (nv) | **FFM%  T0**  (nv) | **Initial MRI** | **Follow-up MRI** |
| --- | --- | --- | --- | --- | --- |
| 1 | -1.1 | 19.4  (17) | 80.6  (84) | 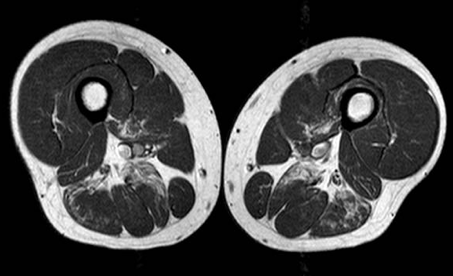 | 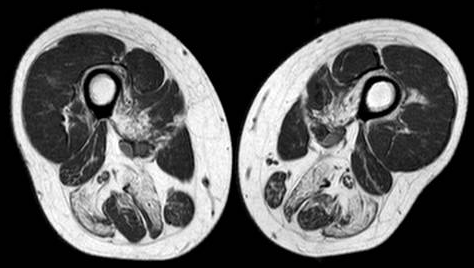 |
| 2 | -0.6 | 19.7  (18) | 80.3  (82) | 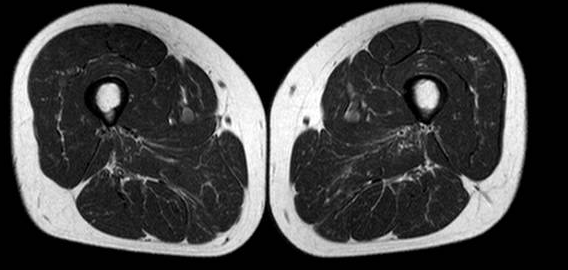 | 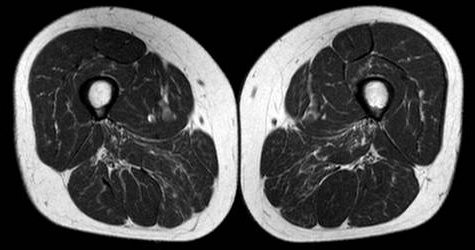 |
| 3 | -1.5 | 9.4  (19) | 90.6  (80) | 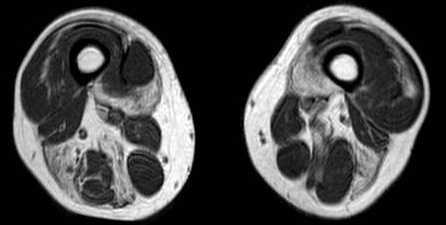 | 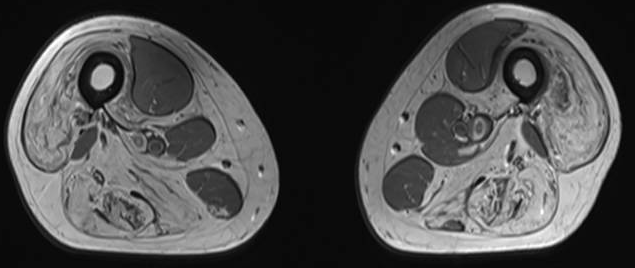 |
| 4 | -1.11 | 26  (19) | 74  (77) | 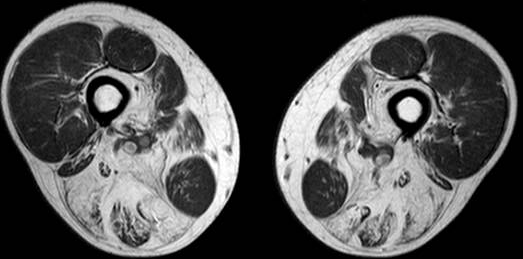 | 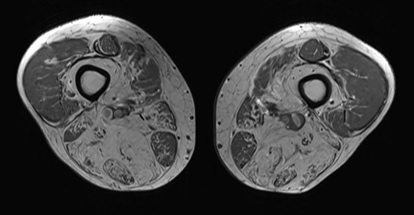 |
| 5 | -2 | 26.3  (22) | 73.7  (76) | 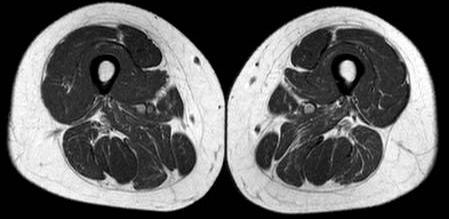 | 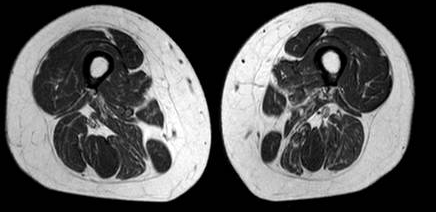 |
| 6 | -2.2 | 50.5  (21) | 49.5  (77) | 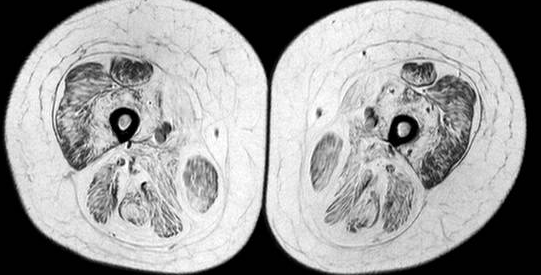 | **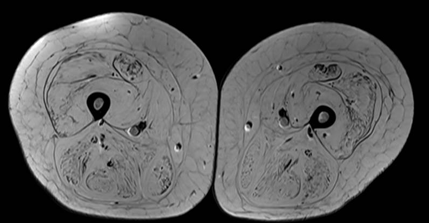** |
| 7 | -0.4 | 29.2  (24) | 70.8  (77) | 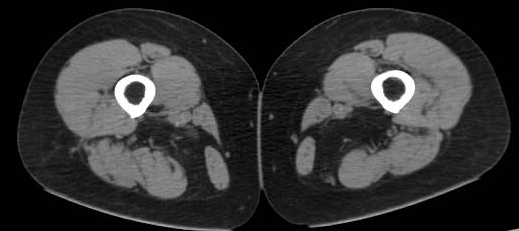 | 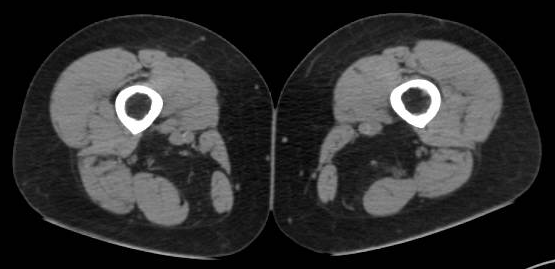 |
| 8 | -0.3 | 28.3  (22) | 71.7  (70) | 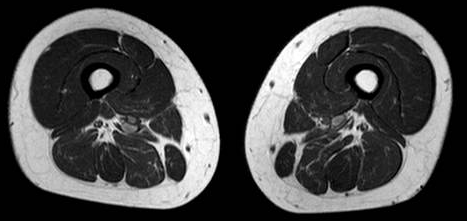 | 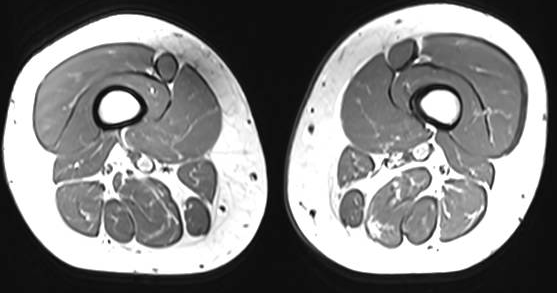 |
| 9 | -0.3 | 25.3  (24) | 74.7 (64) | 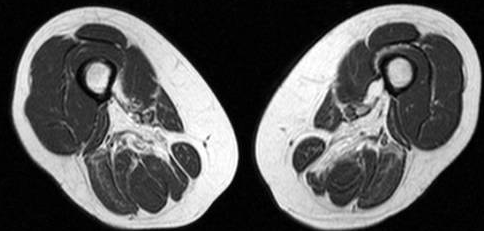 | 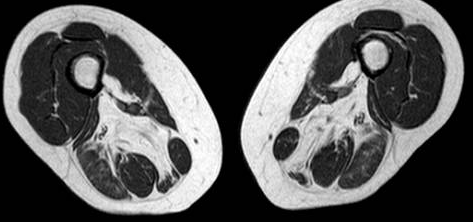 |
| 10 | -1.8 | 36.7  (24) | 63.3  (65) | 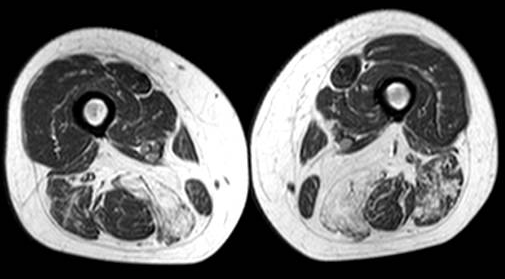 | 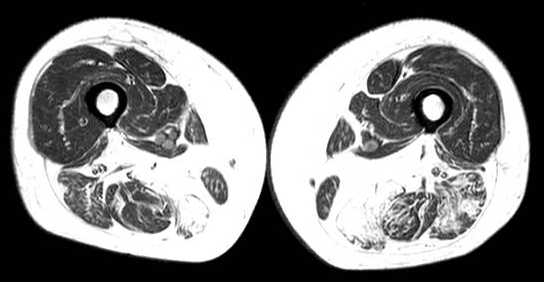 |
| 11 | -0.5 | 34  (23) | 66  (71) | 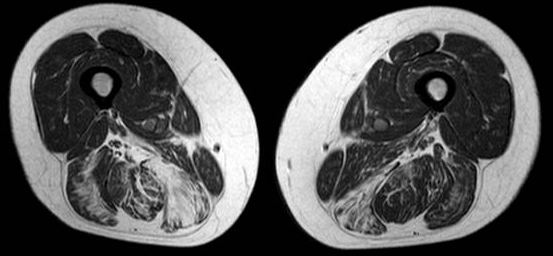 | 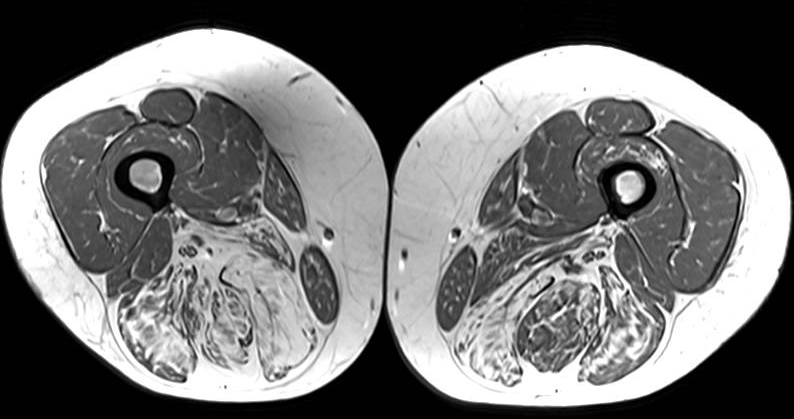 |
| 12 | -1 | 12  (23) | 88  (74) | 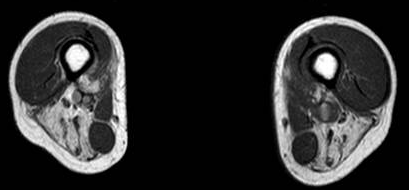 | 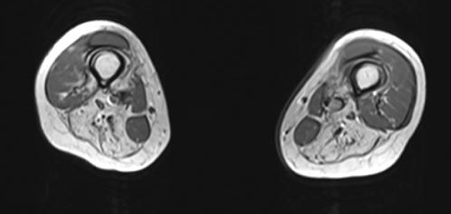 |
| 13 | -0.9 | 7.8  (16) | 92.2  (88) | 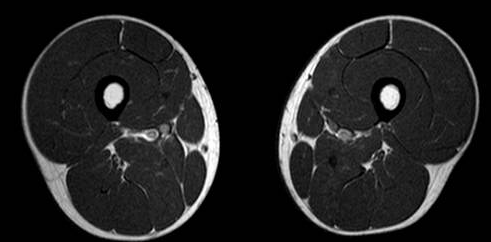 | 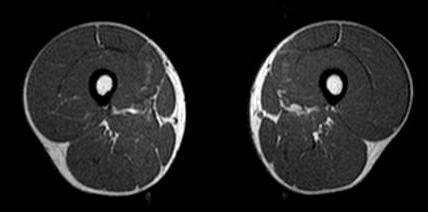 |
| 14 | -0.6 | 24.7  (20) | 75.3  (75) | 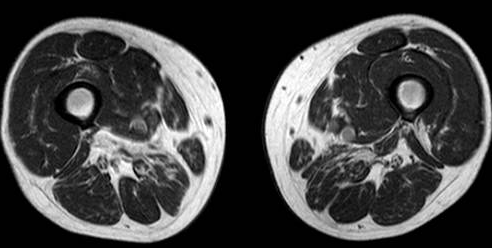 | 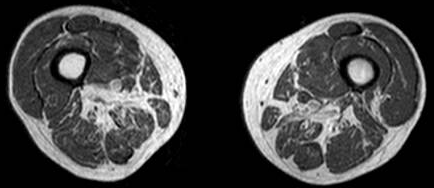 |
| 15 | -0.3 | 8.2  (17) | 91.8  (84) | 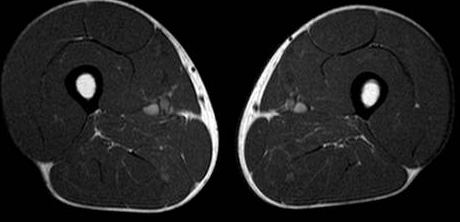 | 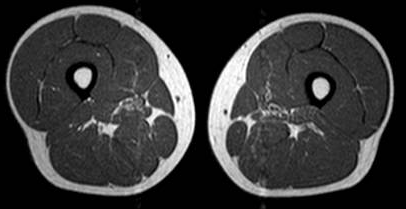 |

nv = normal value matched for age, height and sex

Patient 3 and 4 show severe variation in muscle fatty degeneration but not in muscle function.

*Optimal fat levels by age and sex (overall: FM >20% for males and >25% for females)
